# Editorial: Pushing the limits of motor function recovery in rehabilitation: Basic to applied research based on neuroscience

**DOI:** 10.3389/fnhum.2023.1160632

**Published:** 2023-02-23

**Authors:** Hideki Nakano, Akiyoshi Matsugi, Tomotaka Ito, Kosuke Oku, Masahiro Sakita

**Affiliations:** ^1^Graduate School of Health Sciences, Kyoto Tachibana University, Kyoto, Japan; ^2^Faculty of Rehabilitation, Shijonawate Gakuen University, Daito, Japan; ^3^Faculty of Rehabilitation, Kawasaki University of Medical Welfare, Kurashiki, Japan

**Keywords:** rehabilitation, neuroscience, neurophysiology, neural plasticity, motor recovery, basic research, clinical research, advanced technology

Functional recovery plays an essential role in maximizing the motor function of patients undergoing rehabilitation. However, functional recovery depends on the patient's condition, site of injury, and degree of damage. In addition, a certain level of functional recovery may be achieved but may then stagnate. The limitations of motor function recovery in rehabilitation have changed dramatically in recent years through neuroscientific findings and advanced technologies.

In this editorial, we focus on basic and applied neuroscience-based research aimed at pushing the limits of motor function recovery during rehabilitation. Seven submissions were collected from internationally renowned researchers in this field: four original papers, two systematic reviews, and one clinical trial.

In the field of rehabilitation medicine, electrical stimulation (ES) has conventionally been used for a wide range of clinical applications, including transcranial ES to modulate brain activity and peripheral ES to reduce pain/spasticity or increase muscle strength. The usefulness of ES as adjunctive therapy is well known. Mitsutake et al. and Nishi et al. conducted basic or clinical research using ES. Their findings extend the understanding of the novel vestibular intervention method, noisy galvanic vestibular stimulation (nGVS), and postural control (Iwasaki et al., 2014) and highlight the possibilities of transcutaneous electrical nerve stimulation (TENS).

Mitsutake et al. conducted an exploratory study that examined the effects of nGVS on postural control using kinematic parameters such as muscle activity and joint motion in the lower extremities. They found that nGVS reduced ankle joint motion during postural control, especially when standing on an unstable surface with eyes closed when compared to with eyes open. Their findings suggest the need to elucidate the effects of nGVS on postural control in terms of not only sensory strategies, but also physical responses. Further research on this topic may clarify the factors of nGVS that improve balance.

Although many patients with spinal cord dysfunction experience dysesthesia (Finnerup et al., 2001), an effective and systematic rehabilitation approach for dysesthesia has not been established. Nishi et al. developed a novel TENS method that synchronized stimulus intensity and frequency with the somatosensory profile of a patient's dysesthesia and verified its immediate effects. They reported that the novel TENS method resulted in a significant improvement in dysesthesia when compared to the conventional TENS method. This novel method is expected to contribute to breakthrough interventions for patients with recalcitrant dysesthesia.

Stroke and spinal cord injury cause gait dysfunction, which is a major factor in the decline of quality of life and activities of daily living (Takashima et al., 2017). Recently, the effectiveness of robot-assisted gait training (RAGT) therapy (Cheung et al., 2017) and non-invasive brain stimulation (NIBS) (Elsner et al., 2020) has been reported in rehabilitation efforts for lower limb motor function, and specific protocols are being considered. Kuwahara et al. showed that, because RAGT and NIBS are based on the principle of activity-dependent neuroplasticity, their combined use may amplify their effectiveness. Moreover, repetitive transcranial magnetic stimulation may be useful as a NIBS for use in combination with RAGT and will be discussed in future research.

Amelioration of balance and walking in post-stroke individuals is of paramount concern in rehabilitation (Park and Kim, 2019). A meta-analysis conducted by Yin et al. revealed that whole-body vibration training exerted a salutary effect on balance and training in patients after stroke. Additionally, the study determined that training is a safe and efficacious method for restoring walking dysfunction in these patients. This is a seminal finding that may abridge the time until post-stroke patients can return to their homes and communities and enhance their overall quality of life. Whole-body vibration training is expected to be widely implemented in clinical settings as a form of rehabilitation therapy.

One of the neuromuscular diseases with an imbalance of postural control is myotonic dystrophy type 1 (DM1) (Harley et al., 1993). Scarano et al. reported that the sensory organization test and motor control test paradigms of equi-test posturography were used to evaluate static balance in patients with DM1. This indicates that participants with DM1 depended on the visual rather than somatosensory and vestibular systems, suggesting that frequent falls in DM1 are not only due to muscle weakness. This finding can provide further insight into the possibility of sensory reorganization training to improve balance and prevent falls in patients with DM1.

To improve balance and gait ability in patients with neuromuscular disease, exoskeletons (Siviy et al., 2022) for the lower limbs have been developed and used worldwide. However, the exoskeleton itself physically interferes with postural control. Therefore, for an exoskeletal support device to properly support joint motion, its postural control characteristics must be known upon arrival. Layne et al. reported postural sway and electromyographic activity in lower limb muscles during static motion and perturbation with and without the use of an exoskeleton in a healthy population. This study revealed that the exoskeleton slightly affects the speed of body sway in a similar manner, and the effects on muscle activity are different for each individual. This indicates that exoskeletons must be personalized to meet the specific capabilities and needs of each individual end user for neurorehabilitation.

Repetitive peripheral magnetic stimulation (rPMS) is a noninvasive method of inducing muscle contraction by applying magnetic fields to the peripheral nerves. Intrinsic sensory inputs from proprioceptors located in joints, muscles, tendons, and skin play a crucial role in the formation of body representations (Naito et al., 2016). Xia et al. employed rPMS to demonstrate the potential of body representations in reaction to wrist proprioceptive acuity. The reconstitution of altered body representations in the brain and body dysfunction are critical. The outcomes of this study imply that this discovery may be significant for the evaluation and treatment of rehabilitation aimed at reconfiguring body representations.

Finally, the articles published on this Research Topic contain numerous new findings that provide hints for pushing the limits of motor function recovery in rehabilitation. We hope that readers will use these findings to elucidate the neural mechanisms of motor function recovery, develop objective and quantitative evaluation methods, and ensure practical application of optimal rehabilitation.

## Author contributions

Writing—original draft preparation: HN, AM, TI, and KO. Writing—review and editing: HN, AM, TI, KO, and MS. All authors have made a substantial contribution to the work and approved it for publication.
